# The First Scale-Up Production of Theranostic Nanoemulsions

**DOI:** 10.1089/biores.2014.0030

**Published:** 2015-04-01

**Authors:** Lu Liu, Christina Bagia, Jelena M. Janjic

**Affiliations:** ^1^ Graduate School of Pharmaceutical Sciences, Mylan School of Pharmacy, Duquesne University, Pittsburgh, Pennsylvania.; ^2^ Chronic Pain Research Consortium, Duquesne University, Pittsburgh, Pennsylvania.; ^3^ McGowan Research Institute for Regenerative Medicine, University of Pittsburgh, Pittsburgh, Pennsylvania.

**Keywords:** drug development technologies, drug discovery, inflammation

## Abstract

Theranostic nanomedicines are a promising new technological advancement toward personalized medicine. Although much progress has been made in pre-clinical studies, their clinical utilization is still under development. A key ingredient for successful theranostic clinical translation is pharmaceutical process design for production on a sufficient scale for clinical testing. In this study, we report, for the first time, a successful scale-up of a model theranostic nanoemulsion. Celecoxib-loaded near-infrared-labeled perfluorocarbon nanoemulsion was produced on three levels of scale (small at 54 mL, medium at 270 mL, and large at 1,000 mL) using microfluidization. The average size and polydispersity were not affected by the equipment used or production scale. The overall nanoemulsion stability was maintained for 90 days upon storage and was not impacted by nanoemulsion production scale or composition. Cell-based evaluations show comparable results for all nanoemulsions with no significant impact of nanoemulsion scale on cell toxicity and their pharmacological effects. This report serves as the first example of a successful scale-up of a theranostic nanoemulsion and a model for future studies on theranostic nanomedicine production and development.

## Introduction

A growing number of nanosystems is reported in recent literature as theranostic nanomedicines.^1^ These nanosystems are aimed to fulfill multiple roles: image disease state and therapeutic response, provide targeted drug delivery, control drug release, and image drug delivery efficacy.^2–4^ Such a multifunctional nanosystem design, however, increases preparation complexity, posing significant challenges for future production on a clinical scale and quality control. Consequently, this can slow down theranostic nanomedicine clinical translation and also impact pre-clinical research in animals. This is especially of concern as theranostic nanomedicines move into biomedical research as disease pathway probes or adjuvant therapies for cancer, inflammation, and other chronic diseases. Furthermore, theranostic nanomedicines offer unique opportunities for regenerative medicine as they can be applied into clinical use as imaging-supported delivery systems or tracking devices for therapeutic cells.^1,5^ For all theranostic nanomedicine applications, the reproducibility of animal data is directly related to their quality. Most pre-clinical reports on theranostics do not discuss, or discuss in very limited amount of detail, how much theranostic formulation was produced for animal testing. The goal of this study is two-fold: (1) draw attention to the need for further research on scalable processes for production of theranostic nanosystems; and (2) showcase one successful example where theranostic nanoparticles were produced on scale-up to 1,000 mL. To achieve these goals, we selected for this scale-up study one of our earlier reported theranostic nanoemulsions with celecoxib as a model poorly soluble drug.^6^

Nanoemulsions are kinetically stable emulsions with a droplet size typically between 100 and 500 nm, high oil content, and low amounts of surfactants.^7^ Nanoemulsions can be applied to increase solubility and bioavailability of poorly soluble drugs^8–12^ used as is or incorporated into other dosage forms such as capsules and gels.^12–14^ They are produced by high-energy processing (microfluidization and sonication)^15^ and low-energy emulsification methods.^16^ The focus of this report is on perfluorocarbon (PFC) nanoemulsions, which are currently extensively investigated for diverse biomedical applications, such as MRI,^5,17–19^ ultrasound^20^ and photoacoustic imaging,^21^ oxygen delivery,^22,23^ and image-guided drug delivery.^20^ PFCs are highly biologically inert chemically stable materials that can be quantitatively detected *in vivo* by ^19^F MRI.^18,24^ PFC nanoemulsions are selective inflammation imaging agents.^25–28^ They are also an attractive platform for nanomedicine. PFC nanoemulsions can deliver the antigen to dendritic cells (DCs), boosting the immune response in DC-based vaccines,^29^ or deliver antiproliferative drugs.^30^ Although there are numerous reports on producing PFC nanoemulsions, the manufacturing process development and scale-up are not fully investigated.

In this study, we present the first drug-loaded PFC nanoemulsion (theranostic) produced on scale, quality assessments, and comparative *in vitro* data between small and large scales. In the previous study, we have produced, on a small scale (25 mL), the near infrared (NIR)-labeled perfluoropolyether (PFPE) nanoemulsion validated for stability, imaging properties, and anti-inflammatory action *in vitro*.^6^ In this study, this nanoemulsion was prepared on three levels of scale: small (54 mL), medium (270 mL), and large (1,000 mL). The prepared nanoemulsions were evaluated in detail *in vitro* for colloidal properties, stability, and in cells. Specifically, we tested nanoemulsion long-term stability upon storage for 90 days, stability when exposed to select stress tests, and evaluated for cellular toxicity and pharmacological effects *in vitro*. Our data demonstrate that scale-up of a celecoxib-loaded theranostic nanoemulsion is feasible and nanoemulsion quality is maintained across all scale levels tested. We hope that following this successful example of theranostic nanomedicine scale-up and manufacturing, more studies will follow leading to successful pharmaceutical development of theranostics and their clinical translation.

## Materials and Methods

### Materials

Pluronic^®^ P105 was obtained from BASF Corporation. Cremophor EL (CrEL) was purchased from Sigma Aldrich. Miglyol 812N, Dulbecco's modified Eagle's medium (DMEM), fetal bovine serum (FBS), and the Raw 264.7 cell line were purchased from ATCC. Prostaglandin E_2_ EIA Kit-monoclonal was purchased from Cayman Chemical Company. DiR dye was from Life Technologies. Perfluoro(polyethylene glycol dimethyl ether) or PFPE oxide (CF_3_O(CF_2_CF_2_O)_n_CF_3_, where *n*=8–13) was obtained from Exfluor Research Corporation.

### Equipment

Microfluidizer M110S and Microfluidizer M-110EH-30 from Microfluidics Corporation, Westwood, Massachusetts, were used for all nanoemulsion preparations.

### Preparation of CrEL/P105 surfactant mixture

The CrEL and pluronic P105 surfactant mixture was prepared as reported earlier.^6^ Briefly, the solutions of the surfactants were prepared separately: 4 g of P105 and 6 g of CrEL were dissolved in two 100-mL volumetric flasks filled with deionized water (DI-H_2_O). The two solutions were mixed at a 1:1 v/v ratio. The mixture is then placed in a water bath at 45°C, rotated at a speed of 80 for 20 min, and then chilled on ice for 15 min. The resultant mixed surfactant solution was refrigerated at 4–10°C until use.

### Preparation of nanoemulsions with Microfluidizer M110S (small scale)

The preparation of the small-scale nanoemulsions (Table 1) is based on the already published formulations.^6,31^ Briefly, Miglyol 812N and DiR dye stock solution were mixed and stirred at 350 rpm for 15 min, then PFPE oxide was added, and the solution was stirred for 15 min more. The surfactant mixture (P105/CrEL) was then combined and stirred for 15 min at the same speed. The final solution was then placed into an ice bath and sonicated for 1 min, and then microfluidized on a precooled Microfluidizer M110S for 10 passes (operating liquid pressure ∼17,500 psi). For the celecoxib-loaded nanoemulsion, the drug was predissolved in Miglyol 812N overnight and the DiR dye stock solution was prepared in ethanol (2 mM).

**Table 1.** **Nanoemulsion Scale-Up Formulations**

Components^a^	A^b^	B^b^	C	D	E	F
Miglyol 812N	4	4	20	20	74	74
PFPE oxide	4	4	20	20	74	74
P105/CrEL 2:3 (5% w/v)	46	46	230	230	852	852
Celecoxib (mg)	—	10.3	—	51.6	—	190.9
Total volume	54	54	270	270	1,000	1,000

^a^ Values represent mL of liquid.

^b^ For the formulations A and B, DiR dye was incorporated.

### Preparation of nanoemulsions with Microfluidizer M-110EH-30 (medium and large scale)

For the scale-up experiments (Table 1), we used a high shear fluid processor M-110EH-30. More specifically, for the medium scale (270 mL), Miglyol 812N and PFPE oxide were mixed and stirred at 350 rpm for 15 min, the surfactant mixture (P105/CrEL) was added, and the solution was stirred for 15 min more at the same speed while it was placed into an ice bath. The pre-emulsification solution was sonicated for 2 min while stirring at 4°C. Afterwards, the solution was microfluidized on a precooled Microfluidizer M-110EH-30 for five passes (operating liquid pressure ∼15,000 psi). For the large scale (1,000 mL), Miglyol 812N and PFPE oxide were mixed and stirred at 350 rpm for 15 min, the surfactant mixture (P105/CrEL) was added, and the solution was stirred at higher speed (600 rpm) for 30 min in an ice bath. The pre-emulsification solution was sonicated for 5 min while stirring at 4°C. Afterwards, the solution was microfluidized on a precooled Microfluidizer M-110EH-30 for three passes (operating liquid pressure ∼15,000 psi). For all the experiments, temperature was controlled for each step and celecoxib was predissolved in Miglyol 812N overnight.

### DLS measurements for nanoemulsions

Dynamic light scattering (DLS) of all nanoemulsion samples collected in stability studies was performed as earlier reported.^6,31^ Nanoemulsion samples were prepared by dilution in deionized water or other media at 1:40 v/v.^6^ All samples were analyzed at 25°C with a scattering angle of 173° on Zetasizer Nano ZS (Malvern Instruments).

### Stability assessment under filtration and centrifugation

We determined the size distribution of the nanoemulsion droplets before and after filtration. Samples (10 mL volume) of undiluted nanoemulsions were filtered using 0.22-μm syringe filters (Millex^®^-GS, Merck Millipore Ltd., MF-Millipore™ membrane). DLS measurements were taken after the nanoemulsion samples were diluted in water (1:40 v/v) and allowed to equilibrate at room temperature for at least 30 min before each measurement. All DLS measurements were done at room temperature.^6^ We also determined the size distribution of the nanoemulsion droplets before and after centrifugation. The nanoemulsions were diluted (1:40 v/v) in water, serum-free DMEM, 10% FBS DMEM, or 20% FBS DMEM. Diluted nanoemulsions were centrifuged using Centrifuge 5804R (VWR; Eppendorf AG) at 1,100 rpm for 10 min. The samples were then transferred to DLS cuvettes, and measurements were taken after equilibrating at room temperature for at least 30 min before each measurement. All measurements were done at room temperature.^6^ Samples were analyzed at 25°C with a scattering angle of 173°.

### Stability assessment in cell culture media

Furthermore, the colloidal stability of nanoemulsions in biological media was also evaluated by measuring the droplet diameter. More specifically, for these stress studies, nanoemulsions were incubated at 37°C (1:40 v/v) in deionized water and cell culture medium (serum-free DMEM, 10% and 20% FBS DMEM) for up to 72 h. Undiluted samples were measured for droplet size, polydispersity, and zeta potential at 25°C with a scattering angle of 173° on Zetasizer Nano.

### pH measurements

The pH values of the nanoemulsions were measured at ambient temperature using a double-junction, glass body refillable pH electrode (Oakton) attached to an Oakton pH meter 1100 series. Before taking any measurements, the pH meter was calibrated using two standard buffers from Fisher Scientific at pH 4.00 (certified pH 3.99–4.01 at 25°C) and 7.00 (certified pH 6.99–7.01 at 25°C).

### Cell culture

#### Cell viability

Cell viability was assessed using CellTiter-Glo^®^ luminescence assay as reported earlier.^6^ Briefly, mouse macrophages (Raw 264.7) were seeded in a 96-well plate at 10,000 cells per well. After overnight incubation (18–20 h) at 37°C and 5% of CO_2_, culture media were removed and adherent cells were exposed for 24 h to nanoemulsions A, B, C, D, E, and F (prediluted in fresh, warm complete media) at different nanoemulsion dilutions (0–79.4 μL of nanoemulsion/1 mL of media). Upon completed incubation, 40 μL of CellTiter-Glo reagent was added to each well. Plates were covered with aluminum foil to protect samples from light and mixed on Lab Doctor™ Orbital Shaker at the speed of 70 for 20 min. Luminescence was recorded on a microplate reader (1420 Multilabel Counter, Victor^3™^; Perkin Elmer).

#### PGE_2_ assay

To investigate the *in vitro* therapeutic efficiency of the drug carrier for the scale-up nanoemulsions, the effect of these nanoemulsions on prostaglandin E_2_ (PGE_2_) production by macrophages was assessed by comparing the effect on PGE_2_ production with free drug (celecoxib solution in DMSO). RAW 246 cells were seeded in a six-well plate at 0.8 million cells per well and incubated overnight. We expose the cells with nanoemulsions E and F at 1.4 mg/mL PFPE concentration (9.28 μM celecoxib), free drug dissolved in DMSO (9.28 μM), and DMSO for 24 h. Fresh media were added to unexposed cells. After overnight incubation, all cells were washed with DPBS (2×). Bacterial toxin lipopolysaccharide (LPS) at 500 ng/mL in the full culture media was added to each well (2 mL in each well) with exposed and unexposed cells incubating for 4 h. Unexposed cells treated with LPS were designed as control, and unexposed cells without LPS stimulation were designed as untreated. After 4 h of incubation, supernatant was collected and analyzed using the commercially available PGE_2_ ELISA kit. Samples were analyzed at two different dilutions (1:5 and 1:10) and triplicates of each dilution were used. Assessment of PGE_2_ production in the supernatant and data analysis were performed according to the manufacturer's instructions.^6^

## Results and Discussion

In this report, we focused on demonstrating the feasibility of producing theranostic nanoemulsions on scale. We also explored the effects of microfluidization instrumentation used on the nanoemulsion product quality. Nanoemulsions, produced on three scales, were evaluated by a combination of measurements, including DLS for size distribution and zeta potential and pH measurements for stability. Stability assessments were performed upon storage for up to 90 days and in biologically representative media for up to 72 h. We also evaluated nanoemulsions for their effects on model inflammatory cells *in vitro*. Cell toxicity was tested with nanoemulsion effect on COX-2 in activated macrophages. The presented data indicate a high level of processing robustness and stability of produced nanoemulsions.

**FIG. 1.**
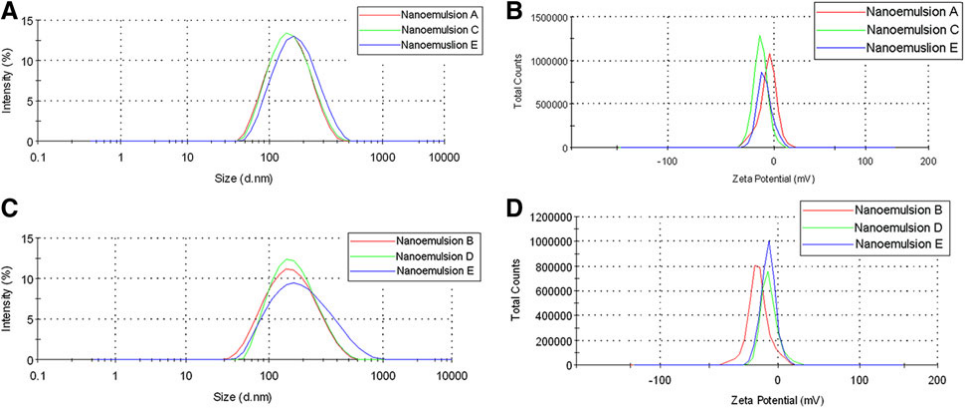
Size distribution and zeta potential comparison between small (54 mL, nanoemulsions A and B), medium (270 mL, nanoemulsions C and D) and large scale (1,000 mL, nanoemulsions E and F). **(A)** Size distribution and **(B)** zeta potential comparison for nanoemulsions without the drug; **(C)** Size distribution and **(D)** Zeta potential comparison for nanoemulsions with the drug.

**FIG. 2.**
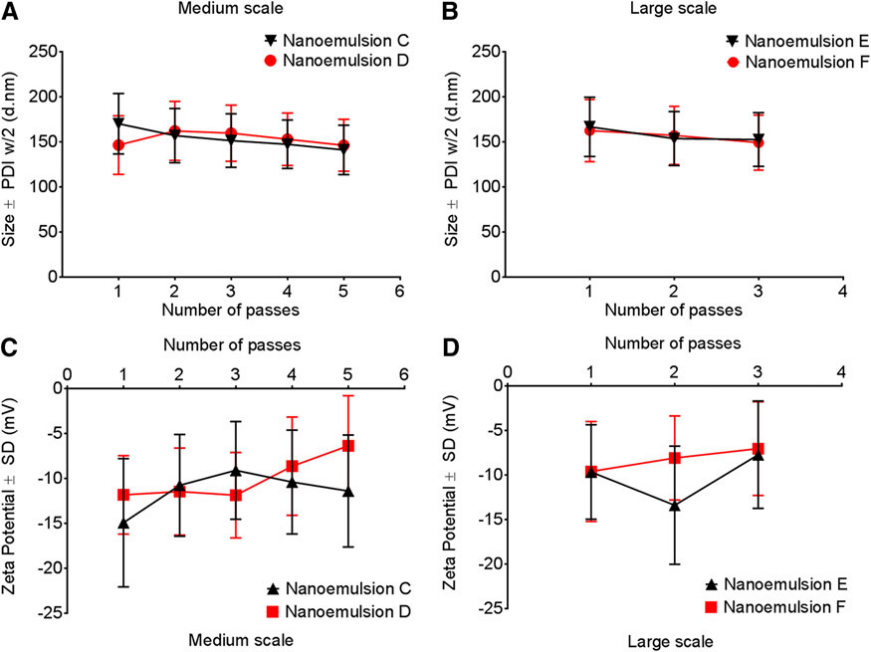
Effect of number of passes on size, polydispersity, and zeta potential for medium- and large-scale nanoemulsions. **(A)** Size and polydispersity of nanoemulsions without drug, nanoemulsion C, and with drug, nanoemulsion D, on medium scale 270 mL; **(B)** Size and polydispersity of nanoemulsions without drug, nanoemulsion E, and with drug, nanoemulsion F, on large scale 1,000 mL; **(C)** Zeta potential for medium-scale nanoemulsions C and D; **(D)** Zeta potential for medium-scale nanoemulsions E and F.

Reported theranostic nanoemulsions are produced by microfluidization at three different scales, small (54 mL), medium (270 mL), and large (1,000 mL) (Table 1). Our earlier reported methods for PFC nanoemulsion preparation were adapted to accommodate the increase in processing volume and change of instrumentation.^6,31^ The small-scale nanoemulsions were processed on the Microfluidizer M110S, and the medium- and large-scale nanoemulsions were processed on the Microfluidizer M-110EH-30. Our DLS measurements indicated that the nanoemulsion particle size distribution and zeta potential distribution were not significantly affected by the nanoemulsification scale or instrument used (Fig. 1). Furthermore, the number of passes was progressively decreased as the scale was increased to prevent product heating.^32^ The nanoemulsion temperature at the small scale in between passes was ∼8–10°C, but when the production was scaled up, the temperature was elevated to ∼23–24°C. This temperature increase did not affect the quality of the final products, as indicated in Figure 1. No change in size or polydispersity index (PDI) was observed by decreasing the number of passes between small (10 passes), medium (5 passes), and large scale (3 passes) (Fig. 2). These observations are significant because they indicate robustness of the method and allow for savings in processing time, which further increases applicability to theranostics loaded with temperature or shear-sensitive drugs and/or imaging moieties. These results strengthen the argument for decreasing the number of passes during the manufacturing process, which can lead to decreased production costs.

**FIG. 3.**
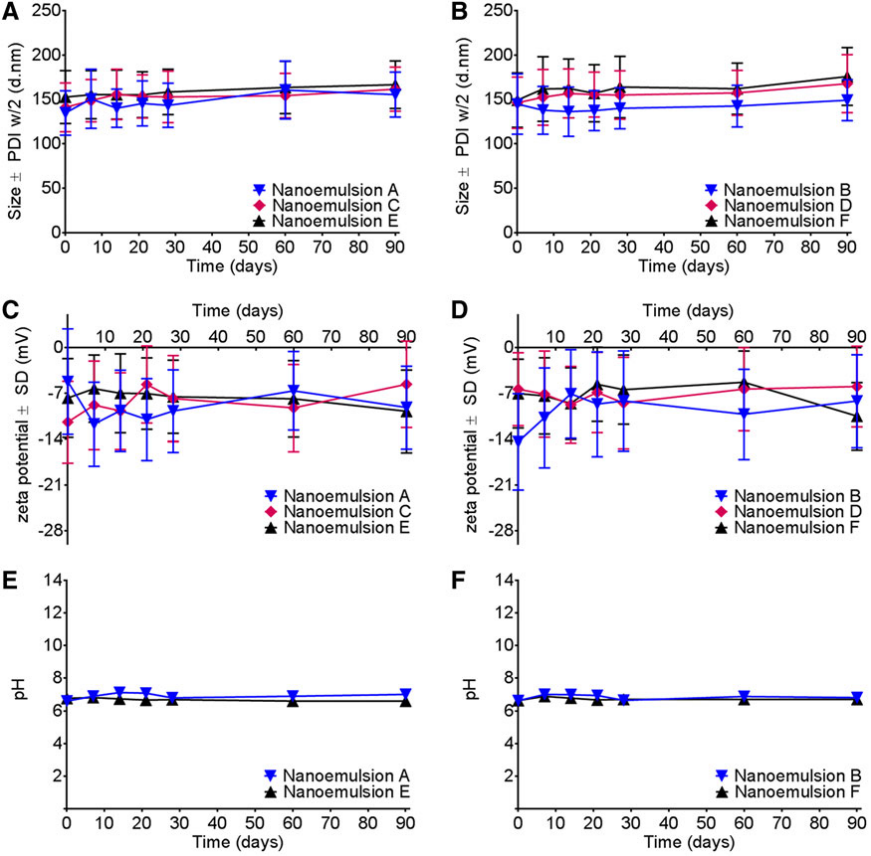
Nanoemulsion stability evaluations. Effects of scale on particle size and zeta potential measurements and pH upon storage at 4°C for 90 days. **(A)** Size measurements of nanoemulsions (A, C, and E) without drug; **(B)** Size measurements of drug-loaded nanoemulsions (B, D, and F); **(C)** Zeta potential measurements of nanoemulsions (A, C, and E) without drug; **(D)** Zeta potential measurements of drug-loaded nanoemulsions (B, D, and F); **(E)** pH measurements of nanoemulsions A and E without a drug; **(F)** pH measurements of drug-loaded nanoemulsions B and F.

**FIG. 4.**
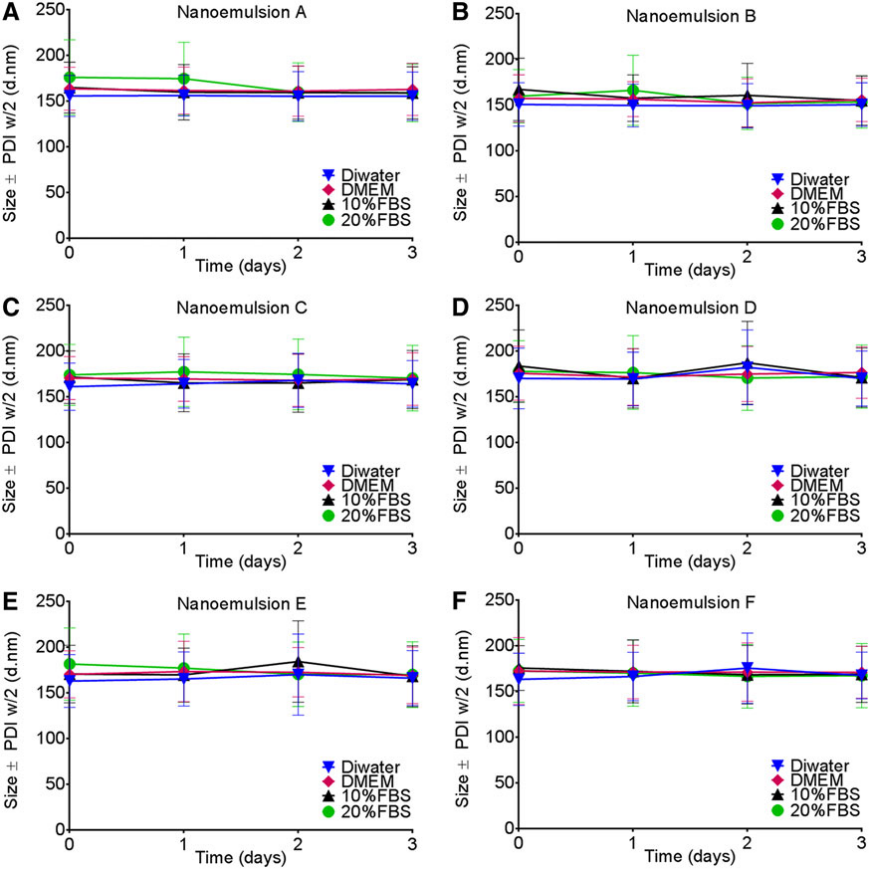
Nanoemulsion stability in biological media measured by dynamic light scattering (DLS) for small-scale nanoemulsions with/without a drug **(A, B)**, medium scale **(C, D)**, and large scale **(E, F)**. Nanoemulsions were diluted at 1/40 (v/v) in deionized water, serum-free Dulbecco's modified Eagle's medium (DMEM) cell culture media, 10% fetal bovine serum (FBS), and 20% FBS DMEM and incubated for up to 72 h at body temperature (37°C). Samples were tested by DLS for size and polydispersity at times 0, 24, 48, and 72 h. Data represent average droplet size (Z-average) with standard deviation as half of polydispersity width. Incubation samples were measured by DLS, undiluted.

**FIG. 5.**
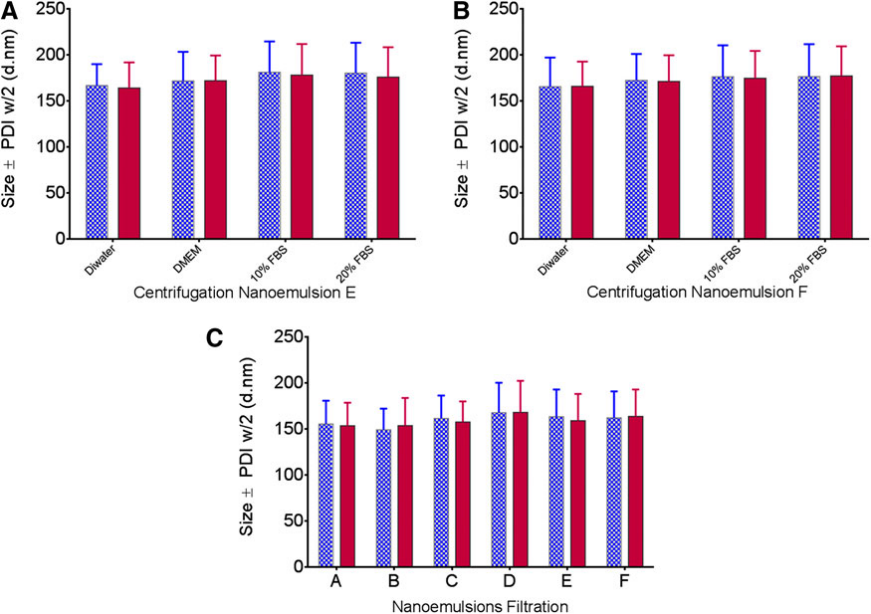
Centrifugation stability of large-scale nanoemulsions E **(A)** and F **(B)**; filtration stability for nanoemulsion A, B, C, D, E, and F **(C)**. In all plots, standard deviation represents polydispersity width half (PDIw/2), indicating the size distribution for each nanoemulsion under testing conditions. Blue open bars represent measurements before the stress induction, and solid red bars are measurements after the stress was applied (centrifugation or filtration).

Furthermore, our measurements indicated that the presence of the drug in produced nanoemulsions did not affect size, zeta potential, or pH (Fig. 3) by production of three different scales and using two types of microfluidizers. Applicability of theranostic nanoemulsions in advanced preclinical testing and clinical trials in the future relies on their quality. To evaluate their shelf life, we tested all produced nanoemulsions for droplet size, polydispersity, zeta potential, and pH at storage conditions (4°C) for 90 days. Figure 3A and B shows no significant change in droplet size after 90 days of follow-up for nanoemulsions with and without a drug at all levels of the scale (small, medium, and large). Zeta potential was also maintained during the 90-day follow-up at around −7 mV, which further supports the stability of nanoemulsions (Fig. 3C, D). Furthermore, the nanoemulsion pH remained stable at ∼6.8 (Fig. 3E, F). Once nanoemulsions are used *in vivo*, they come into contact with complex biological fluids. To model these stressors, we evaluated their colloidal stability in model biological media (FBS-containing cell culture media) at an elevated temperature (37°C) for 72 h. No significant changes in size and polydispersity were observed upon 72 h of incubation. This suggests that all nanoemulsions (small, medium, large scale) with or without the drug are not affected by the presence of protein, salts, or nutrients (Fig. 4A–F). To further investigate nanoemulsion stability under mechanical stress and the potential impact of the scale of production on nanoemulsion quality, we performed centrifugation and filtration stability tests. It was found that upon centrifugation at 1,100 rpm for 5 min at room temperature and in different media (water, serum-free, and serum-containing cell culture media), the largest scale nanoemulsions (E and F) showed no significant changes in size and polydispersity (Fig. 5A, B). When the nanoemulsions were tested against filtration through a 0.22-μm filter, all nanoemulsions (small, medium, large scale) prepared with or without the drug showed no changes in size and polydispersity (Fig. 5C). These data suggest that all nanoemulsions reported here at three levels of scale remain highly stable when exposed to stressors during storage and use, as we earlier reported for small-scale preparations.^6,31^

**FIG. 6.**
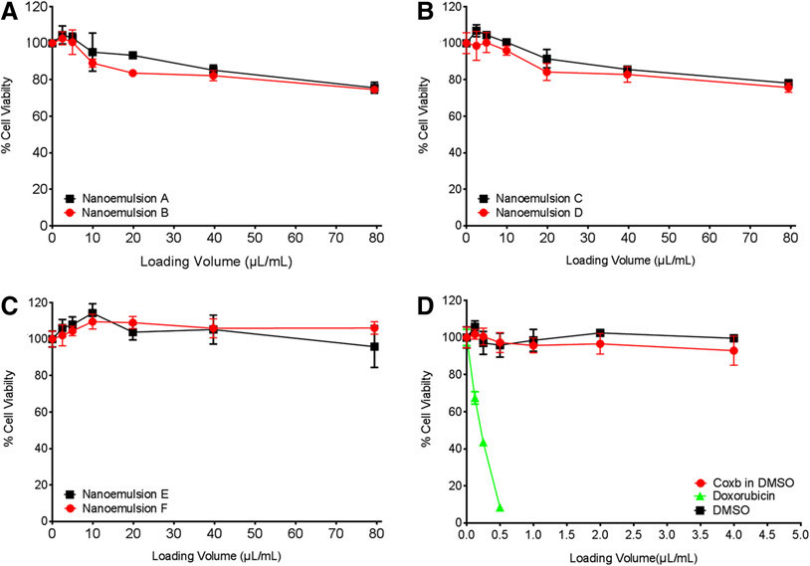
Cell viability testing for nanoemulsions produced at three levels of scale (small, medium, and large). **(A)** Cell viability percentage of small-scale nanoemulsions A and B; **(B)** Cell viability percentage of medium-scale nanoemulsions C and D; **(C)** Cell viability percentage of large-scale nanoemulsions E and F; **(D)** Control cell viability test with free drug (celecoxib in DMSO) at equivalent concentrations to nanoemulsion cell exposures, DMSO (vehicle), and doxorubicin (positive control). All data represent an average±SD (*N*=3).

**FIG. 7.**
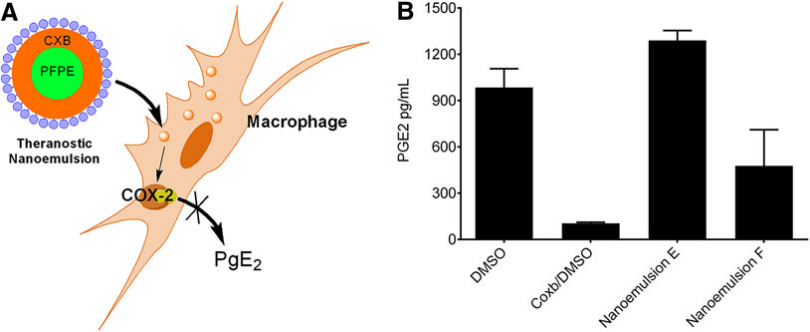
**(A)** Celecoxib-loaded theranostic nanoemulsion inhibits COX-2 enzyme in lipopolysaccharide (LPS)-activated macrophages; **(B)** prostaglandin E_2_ (PGE_2_) release inhibition from LPS-activated macrophages exposed to celecoxib-loaded nanoemulsion F and drug-free nanoemulsion E. Free drug (celecoxib, Coxb) and DMSO were used as controls. Data represent the average from three independent measurements (mean±SD).

We also investigated scale-up nanoemulsions in cell-based assays. To evaluate their effects on cell viability, we used the mouse macrophage cell line (RAW 264.7). Upon overnight incubation at increasing concentration (up to 80 μL loading volume of nanoemulsion per 1 mL of media), we found no significant change in cell viability using CellTiter-Glo^®^ Luminescent Cell Viability assay. As a control, we used free drug (celecoxib [Coxb] dissolved in DMSO) at the same concentration levels as in the nanoemulsions, DMSO only as a negative control, and doxorubicin as a positive control. Figure 6 summarizes cell toxicity assays. Finally, we tested the effects of large-scale nanoemulsion on COX-2 enzyme activity in macrophages, following the same experimental set-up as reported earlier.^6^ When exposed to LPS, macrophages upregulate the COX-2 enzyme, which leads to increased production of PGE_2_. As in the previous study, we show here that exposure to celecoxib-loaded nanoemulsion dramatically reduces release of PGE_2_ from RAW 264.7 cells. Figure 7A illustrates the proposed mechanism of theranostic nanoemulsions on the COX-2 enzyme. Largest scale (1,000 mL) drug-loaded nanoemulsion demonstrated significant inhibition of PGE_2_ release compared with vehicle drug-free nanoemulsions (Fig. 7B). Celecoxib in DMSO was used as the positive control. This result demonstrates that we successfully scaled up a theranostic nanoemulsion and maintained its biological safety (Fig. 6) efficacy (Fig. 7).

## Conclusion

In this study, we report for the first time the successful scale of theranostic nanoemulsions. Processing changes such as instrumentation used and processing temperature did not affect the final product quality in respect to size, polydispersity, zeta potential, or pH. Long-term follow-up upon storage shows no change in stability for all nanoemulsions produced regardless of the scale and presence of drug for up to 90 days. Furthermore, cell assays indicated comparable cell viability profiles between nanoemulsions and with earlier reported data. When large-scale (1 L) nanoemulsion was tested for anti-inflammatory effects in a model cell line (mouse macrophages, RAW 264.7), it showed, as expected, inhibition of the cyclooxygenase 2 (COX-2) enzyme leading to reduced PGE_2_ release. Furthermore, we also show high stability of all nanoemulsions produced when subjected to a variety of stress tests, such as exposure to biologically relevant media, filtration, and centrifugation. These results strongly suggest that theranostic nanosystems if designed with the scale in mind can become viable clinical candidates. We hope this report spurs new investigations and pharmaceutical formulations in the field of theranostic nanomedicine. Producing nanomedicines on a sufficient scale and with maintained quality leads to high quality of pre-clinical testing by removing batch-to-batch variability.

## Abbreviations Used

DCs: dentritic cells
DLS: dynamic light scattering
LPS: lipopolysaccharide
PFC: perfluorocarbon
PGE_2_: prostaglandin E_2_
